# A Transfer Hamiltonian Model for Devices Based on Quantum Dot Arrays

**DOI:** 10.1155/2015/426541

**Published:** 2015-03-24

**Authors:** S. Illera, J. D. Prades, A. Cirera, A. Cornet

**Affiliations:** MIND/INUB Departament d'Electrònica, Universitat de Barcelona, C/Martí i Franquès 1, 08028 Barcelona, Spain

## Abstract

We present a model of electron transport through a random distribution of interacting quantum dots embedded in a dielectric matrix to simulate realistic devices. The method underlying the model depends only on fundamental parameters of the system and it is based on the Transfer Hamiltonian approach. A set of noncoherent rate equations can be written and the interaction between the quantum dots and between the quantum dots and the electrodes is introduced by transition rates and capacitive couplings. A realistic modelization of the capacitive couplings, the transmission coefficients, the electron/hole tunneling currents, and the density of states of each quantum dot have been taken into account. The effects of the local potential are computed within the self-consistent field regime. While the description of the theoretical framework is kept as general as possible, two specific prototypical devices, an arbitrary array of quantum dots embedded in a matrix insulator and a transistor device based on quantum dots, are used to illustrate the kind of unique insight that numerical simulations based on the theory are able to provide.

## 1. Introduction

The demand for increasing the integrated density devices has led to the emergency of a whole generation devices based on confined structures. The MOS (metal-oxide-semiconductor) transistor is the archetype of a confined two-dimensional system [1]. Nevertheless, the possibility to enhance this confinement by embedding low-dimensional structures in an insulating matrix has opened new way for further downscaling. Compared to the standard bulk technology, the corresponding devices based on these structures have increased the structural and conceptual complexity. These structures (quantum dots, wires, or layers) can be used in single-electron devices [2], new memory concepts [3], and photo- or electroluminescent devices [4]. Concerning single-electron devices, they are currently conceived to take advantage of tunnel current between quantum states belonging to nanoscale particles [5, 6]. The single-electron devices based on quantum dots (Qds) appear to be potential candidates to improve, to complete, or even to replace the current MOS technology with which they may remain compatible. In order to be able to asses the potentials and capabilities of the various novel devices, a realistic theoretical estimation of the specific device performance is thus highly desirable. Within this context, the simulations of such devices must be performed not only to understand but also to predict experimental behaviors. Moreover, from a physical point of view, we will learn a lot from these simulations if they are independent of high-level experimental parameters (as tunneling rates, defective interfaces,…, etc.) and are based on low-level concrete ones (geometrical data, barrier height…).

Concerning Qds, they are particularly attractive because they possess discrete energy levels and quantum properties similar to natural atoms or molecules due to the strong confinement in all three directions. This fact affects dramatically the electronic transport properties. Until now, research has mostly concentrated on single Qds and many novel transport phenomena have been discovered, such as the staircaselike current-voltage (*I*-*V*) characteristic [7], Coulomb blockade oscillation [8], negative differential capacitance [9], and the Kondo effect [10].

From experimental point of view, rapid progress in microfabrication technology has made possible coupling Qds system with aligned levels [11–13]. In fact, the use of the Coulomb blockade phenomenon in systems made up of combinations of tunnel junctions and semiconductor Qds seems to offer promising perspectives, in particular in non-volatile memory applications and also for single-electron transistor [14]. Moreover, the concept of multi-dot memory using semiconductor nanocrystals embedded in an insulator matrix as floating-gate has already been demonstrated [15] and the quantization effects have been used in self-aligned double-stacked memory to improve the retention time [16].

From a theoretical perspective, researchers have recently paid much attention to electron transport through several Qds, since multiple Qd provides more Feynman paths for the electron transmission [17]. The complexity of structure and physical mechanism and the prominent role of dimensional and quantum effects characterizing the operation of these novel Qds devices preclude the use of standard macroscopic bulk semiconductor transport theory. Many authors have studied the electron transport using NEGFF (nonequilibrium Green functions formalism) [18, 19], taking into account the potential due to the self-charge. However, up to now nobody has done a computation of transport in an extended arbitrary array of Qds using this framework since this approach is usually unfeasible to implement for large systems. On the other hand, rate equation type models used for lasers or light-emitting diodes often offer a satisfactory description of the charge transport. Moreover, this approach presents a more transparent vision of the electron transport. Thus, this model is easier to tinker with, in order to deal with more complicated nanostructures based on Qds.

In this work, we present in full a model based on noncoherent rate equations [20], which is suitable to study the electron transport in Qd arrays. In a previous work [21], a preliminary version of this methodology was presented and used to obtain analytical solutions for electron transport in simple cases. The methodology was also compared with nonequilibrium Green's function calculations, obtaining favorable results [22]. Despite the simplicity of the model, it provided good results and it was also easily scalable.

Now, a complete model to simulate devices based on Qd arrays is presented. This model creates a compact modeling tool to study and simulate the electronic transport as a function of geometrical and basic material parameters, making possible to use it as a device design tool. The theoretical formalism and the assumptions made in the model are thoroughly described. The interaction between Qds and between these and the leads has been introduced by transition rates and capacitive couplings. The local potential effects were computed self-consistently. Inelastic and backscattering effects were neglected. Concerning the transition rates, the use of* ab initio* calculations is shown to be the best way to fully understand the underlaying tunneling physics in nanostructures. However, if first-principles calculations for single tunnel events were implemented, the huge effort required would make the simulation time increase in an unacceptable manner. This impractical computational time forced us to write a compact model with some assumptions and relax the expectation of accuracy when treating with few-electron devices operating through quantum features. Specifically, we used the one-dimensional WKB approximation, which neglects spatial variation of the wavefunction over the nontransport directions to describe the tunnel processes. The hole transport was also introduced obtaining new current terms and realistic expressions for the capacity in bipolar conduction. All of these have been implemented in a computational code conforming a powerful transport simulation tool that allows reproducing, explaining, and predicting the behavior of multiple devices based on Qds. Furthermore, we also present details about the computational implementation. Finally, in order to show the capabilities of the presented methodology, two examples of practical implementations of Qd-based devices were simulated: one single Qd and a multilayer structure that conform the basic building block of future devices based on Qds.

## 2. The Model

As in any device simulation, the ultimate goal of the presented approach is the prediction of the device response of one specific architecture (geometry, material,…, etc.) to a given variation in the external conditions (bias voltage) via the solution of the dynamical equations. First of all, we are going to describe the device architecture and, after that, we write the underlying equations.

### 2.1. The Structure

**Figure 1:**
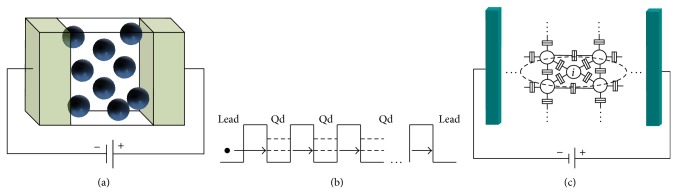
(a) The basic building block that forms the devices based on Qds: an array of Qds (they could be ordered or randomly dispersed) embedded in an insulator matrix sandwiched between two electrodes (leads). (b) Energy band scheme of the structure. The electron starts in the left lead and crosses through the dielectric matrix by tunneling processes assisted by the intermediate Qds. (c) Scheme of the equivalent electrical circuit of the device presented in (a). The Qds (circles) are connected between them and the leads (color blocks) by tunneling junctions. The tunneling junction is described as a capacitor in parallel with a current source.

Figure 1(a) shows the basic building blocks of our device which is in essence an insulator layer sandwiched between two metallic electrodes. Inside the insulator layer, a random distribution of *N* Qds can be inserted. This is the classical structure that is obtained due to the fabrication processes, a superlattice of insulator-semiconductor bilayers. Although a single connected Qd to the leads has been obtained creating a so-called single electron transistor (SET) [29], the research mainstream is focused on the properties of structures with many Qds to create nonvolatile memories [30], light-emitting devices [31], or solar cells devices [32].

Due to the fabrication processes, the insulator thickness is large enough to avoid direct tunneling between the electrodes (or leads). Therefore, the electron current needs to pass through the Qds. In Figure 1(b) the energy band diagram of the system is shown. The Qds are presented as potential wells in the dielectric energy band and they assist the tunneling processes. Thus, a correct description of the tunneling processes among the Qds and Qds-electrodes is needed. These tunneling processes can be described by tunnel junctions. From the electrical point of view, a single tunnel junction is described by a capacitance and a current source that depends on the voltage drop in the junction. The equivalent electrical circuit is shown in Figure 1(c) for an arbitrary Qd array.

From the electrical scheme, two coupling equations govern the response of the system: on one hand we can write the charge conservation in each Qd. In the steady state, the charge conservation for this system is analogous to the electronic Kirchhoff's current law. Thus, using the decomposition of the tunneling junctions in current sources, we can write

(1) $$\begin{array}{c} 0=\underset{j}{\sum }I_{ij}\;i\in N, \end{array}$$

for the *i*th Qd (equivalent to a node), where *N* is the number of Qds and the summation takes into account all the linked elements to this Qd. On the other hand, the current through a junction will depend on the voltage drop in the tunneling junction. Therefore, a second equation is necessary to describe the voltage in each Qd, the same as the Kirchhoff's voltage law. First of all, we are going to show analytical expressions for the tunnel currents through a junctions and, after that, we will calculate the potential in each Qd using the Poisson equation.

### 2.2. Current through a Tunneling Junction

A usual method employed to describe the tunneling processes in devices is the tunneling Hamiltonian approach (also called Transfer Hamiltonian approach). This theory, thoroughly studied by many authors [33–35], treats the tunnelling events as a perturbation. The matrix coefficient |*T*_*LR*_|^2^ quantifies the probability for a particle to transfer from a state of the left side of the barrier to a state of the right side by a tunnel process.

The tunneling rate is determined using time-dependent perturbation theory by considering the electron from one side of the barrier as initial state and the electron on the other side as a final state. The tunneling rate from the left to the right states (both are considered as a part of a continuum) can be calculated using the Fermi's golden rule [36]:

(2) $$\begin{array}{c} d^{2}W_{{\vec{k}}_{L}\to {\vec{k}}_{R}}=\frac{2\pi }{\hbar }{\left|T_{LR}\right|}^{2}\rho_{R}\left(E_{R}\right)\rho_{L}\left(E_{L}\right)\delta \left(E_{R}-E_{L}\right)dE_{R}dE_{L}, \end{array}$$

where *ρ*_*L*_ and *ρ*_*R*_ are the density of states of the left and right side. From this expression, we can see that we only consider ballistic transport. This means that the electron does not suffer energy loss scattering processes when it moves through the barrier. Introducing the energy distribution function in each part of the barrier we can evaluate the total tunneling rate from all occupied states on the left to all unoccupied states on the right [37]:

(3) ΓL→R=∫−∞+∞ρLELfLEL·∫−∞+∞2πħTLR21−fRERhhlh∫−∞+∞2πħ·ρR(ER)δ(ER−EL)dERdEL.

The opposite tunneling rate can be calculated in a similar way. Thus, the net tunneling current *I* = −*q*[Γ_*L*→*R*_ − Γ_*R*→*L*_] assuming symmetry in the transmission coefficient *T*_*LR*_ = *T*_*RL*_ [38] can be written as

(4) $$\begin{array}{c} I_{RL}=\frac{4\pi q}{\hbar }\overset{+\infty }{\underset{-\infty }{\int }}{\left|T_{RL}\left(E\right)\right|}^{2}\rho_{R}\left(E\right)\rho_{L}\left(E\right)\left[f_{R}\left(E\right)-f_{L}\left(E\right)\right]dE, \end{array}$$

where we introduce a factor of 2 to take into account the spin. Therefore, using this approach, we can describe the whole system as independent subsystems (the Qds) connected between them by a transmission probability through the dielectric media. Thus, this methodology allows us to write the currents between the different parts of the system.

In the above expressions, *f*_*R*_ and *f*_*L*_ are the nonequilibrium energy distribution functions in each side of the barrier. These distributions functions take into account how the energy levels are filled (*f*_*R*_) or emptied (1 − *f*_*R*_); as it is expected the electron transport only occurs if the initial state is filled and the final state is empty (see (3)). However, the distribution function of each part of the system is unknown. Assuming that the distribution functions of the electrodes (left *L* and right *R* electrodes) are well described by the equilibrium Fermi Dirac statistics using modified electrochemical potentials, *μ*_*L*_ − *μ*_*R*_ = *qV* where *V* is the applied bias voltage, the problem is reduced to find the nonequilibrium distributions functions of each Qd (*n*_*i*_).

From the definition of the total charge, *N*_*i*_ inside the *i*th Qd can be expressed as

(5) $$\begin{array}{c} N_{i}=\int \rho_{i}\left(E\right)n_{i}\left(E\right)dE. \end{array}$$

Here, we are going to redefine the notation used to describe the distribution functions. The distribution function for each Qd is *n*_*i*_ while we reserve *f*_*L*_ and *f*_*R*_ for the distribution function of the left and right leads, respectively. We can write the evolution charge in time for each Qd as *N*_*i*_ = ∑_*j*_∫*I*_*ji*_ *dt*, where the subscript *j* takes into account all the elements that are linked to the *i*th Qd. Thus, from (5) we can write the evolution of charge in time as a function of the total net current flux for each Qd. This set of integrodifferential equations has a similar form as a usual rate equation. In the following, the different current terms and the elements that appear in (4) are going to be discussed.

#### 2.2.1. Electron and Holes Current Terms

Since the evolution charge in time of each Qd can be written as a function of the net current flux, it is needed to determine all the current contributions. The current contributions can be of two types; the Qds have leads contributions and also neighbors Qd current contributions. These two types of current have the same form in (4), but in each case we need to use the correct distribution function.

**Figure 2:**
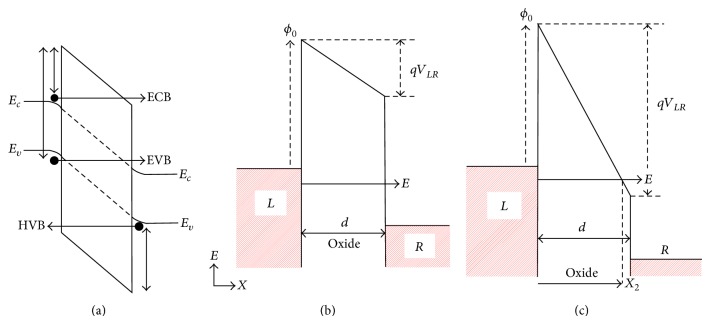
(a) Schematics of the different tunneling processes. Electron from conduction band to conduction band (ECB), electron from valence band to conduction band (EVB), and tunneling from valence band to valence band (HVB) processes. Energy band diagram for the tunneling processes under polarization. (b) If the incident electron energy (*E*) is less than the modified energy barrier (*qϕ*_0_ − *qV*_*LR*_) we use a direct tunnel expression. (c) If the incident electron energy (*E*) is greater than the modified energy barrier (*qϕ*_0_ − *qV*_*LR*_), we use a Fowler-Nordheim expression. These expressions depend on the incident electron energy but also depend on the polarization voltage.

From the point of view of the nature of these contributions, we have three different processes [39]. In Figure 2(a) we show the scheme of the different tunneling processes. The first term corresponds to electron tunneling from conduction band to conduction band (ECB). The second one is an electron tunneling from valence band to conduction band (EVB). Since the transmission coefficients are symmetric, this process also involves the inverse case, tunneling from conduction band to valence band. The last process is related to the holes: hole tunneling from valence band to valence band (HVB).

One important point is how we treat the distinction between electrons and holes. From a physical point of view, the hole conduction can be viewed as electron conduction restricted to the valence band. Therefore, we can consider only electron transport but taking into account the conduction and valence band contributions to the current. Thus, we only need to consider the changing number of electrons in these two bands. Therefore, the time charge evolution equations for each Qd can be written as

(6) dNidt=∫−∞+∞TECB2ρLρiCB(fL−ni)dE+∫−∞+∞THVB2ρLρiVB(fL−ni)dE︸Left  lead  contribution+∫−∞+∞|TECB|2ρRρiCB(fR−ni)dE+∫−∞+∞THVB2ρRρiVB(fR−ni)dE︸Right  lead  contribution,  NeighboringQds  contribution  +∑j,j≠iN∫−∞+∞TECB2ρiBCρjBC(nj−ni)dE+∫−∞+∞THVB2ρiVBρjVB(nj−ni)dE+∑j,j≠iN∫−∞+∞TEVB2ρiBCρjVB(nj−ni)dE+∫−∞+∞TEVB2ρjBCρiVB(nj−ni)dE,

where *i* = 1 ⋯ *N* and we take into account all the contributions for an arbitrary *i*th Qd. The first pair of elements is related to the left lead contribution and the electron and the hole contributions. For simplicity, we assume infinite metallic leads; therefore, we only write the continuum DOS of the leads (*ρ*_*L*_); meanwhile, in the Qd we write the DOS in separate terms, conduction (*ρ*_*i*_^BC^) and valence (*ρ*_*i*_^VB^) bands. In the next section, we will show that the Qds have discrete electron/hole energy levels instead of conduction or valence bands but this expression will not change. Similar contribution is obtained for the right lead. In these two contributions we use the Fermi Dirac distribution function to describe the leads with *μ*_*L*_ − *μ*_*R*_ = −*qV*_*ds*_ electrochemical potentials. In each current term, we use the appropriate transmission coefficient. The last two pairs of current terms represent the current from the neighbor Qds. The subscript “*j*” runs over all the Qds except the Qd that we are considering. In these terms we take into account the different processes, tunneling from the conduction band (CB) to conduction band (CB) and tunneling from valence band (VB) to valence band (VB). We also need to describe the tunneling that mixes the bands (EVB processes) in two ways, from CB to VB and from VB to CB. As it is easy to see, these processes cannot occur at the same time but it is important to take both into account.

The set of equations, (6), can be solved for the steady state. Under our assumption that there is no inelastic scattering, the system can be written in a matrix form and solved for each energy step to obtain the nonequilibrium distribution function for each Qd (*n*_*i*_).

#### 2.2.2. Transmission Coefficients

From (2) the transmission coefficient |*T*_*LR*_|^2^ is defined as the tunnel probability of the electrons crossing through the dielectric media. The tunneling probability is a strong function of the parallel component to the junction interface energy *E*_||_. At each particular total energy *E*, the DOS with a zero *E*_||_ component is heavily weighted by the tunneling probability in (4). Therefore the DOS only takes into account states with *k*_||_ ≈ 0. This approximation may in part be a justification for ignoring the tunneling electron momentum in (3) [40].

Under our assumptions, we use the semiclassical and one-dimensional WKB approximation [36] for the transmission coefficient |*T*(*E*)|^2^:

(7) $$\begin{array}{c} {\left|T\left(E\right)\right|}^{2}=\exp\left\{-\frac{2}{\hbar }\overset{x_{2}}{\underset{x_{1}}{\int }}\sqrt{2m_{\text{diel}}^{*}\left(V\left(x^{\prime }\right)-E\right)}dx^{\prime }\right\}, \end{array}$$

where *x*_1_ and *x*_2_ are the classical turning points, *m*_diel_^*^ is the effective dielectric mass, and *V*(*x*) is the potential barrier of the dielectric material. This barrier is the difference between the bands of the Qd and the dielectric matrix. The transmission coefficients have been derived taking into account the effect of the electric field in the interface, *E*_diel_. The transmission coefficient can be separated in three regions; for incident electrons with less energy than the modified height of the barrier we use a direct tunnel expression. This expression considers that the electrons see a trapezoidal potential barrier. When the incident electrons have energies between the modified height and the total barrier height we use the Fowler-Nordheim expression in which the electrons see a triangular potential barrier. Finally, for incident electrons with energy greater than the barrier we do not assume scattering; therefore, we assign |*T*(*E*)|^2^ = 1. This last case corresponds to elastic transport through the conduction band of the dielectric matrix and only occurs for large bias voltages. The transmission coefficient can be written as

(8) TE2=exp⁡−42mdiel∗3ħqEdielqϕ1−E3/2−qϕ0−E3/2hhhhhhhhhhhhhhhhhhhlhhhhhhhfor  qϕ0≥E,exp⁡−42mdiel∗3ħqEdielqϕ1−E−Ec,13/2hhhhhhhhhhhhhhhhhlhhhhhhhhhfor  qϕ1≥Ehhhhhhhhhhhhhhhhhlhhhhhhhfor  qϕ1hh≥qϕ01hhhhhhhhhhhhhhhhlhhhhhhhhhfor  E≥qϕ1.

The electric field is defined as *E*_diel_ = (*E*_*c*,1_ − *E*_*c*,2_ + *q*Δ*ϕ*)/*qd*, where *qϕ*_1_ is the potential barrier height, *qϕ*_0_ is the modified potential barrier height, *d* is the tunneling distance, *E*_*c*,1_ − *E*_*c*,2_ is *q* times the electrostatic potential between the two elements, and *q*Δ*ϕ* is the work function difference. In Figure 2 a scheme of the barrier is shown under external polarization and the two different tunneling mechanism are also shown.

In a similar way the transmission coefficients for the holes can be derived. In that case, the potential barrier is the difference between the valence bands of the Qd and the dielectric matrix.

#### 2.2.3. Density of States

Another parameter that appears in the expression of the tunneling currents (4) is the density of states (DOS) of both sides of the barrier. As a first order approximation, we propose a simplified model to represent the discrete energy levels in the Qds. We treat each Qd as a finite spherical potential well. The height of the well is the difference between the conduction band energy level of the dielectric matrix and the one of the material that forms the Qd.

Solving the spherical Schrödinger equation inside the well for *l* = 0 we obtain the typical binding states [41]. The number of binding states and their energetically position depend on the height of the well *V*_0_, the radius *R*, and the electron effective mass *m*_Qd_^*^ and *m*_diel_^*^ (mass inside the Qd and in the dielectric media, resp.). Imposing continuity of the wave function and its first derivate in *r* = *R* the equation that determines the binding states is

(9) $$\begin{array}{c} \cot x=-\sqrt{{\left(\frac{\sigma_{0}}{x}\right)}^{2}-\frac{m_{\text{diel}}^{*}}{m_{\text{Qd}}^{*}}}, \end{array}$$

where $\sigma_{0}=\sqrt{(2m_{\text{diel}}^{*}V_{0}/\hbar^{2}){\left(R\right)}^{2}}$ and $x=\sqrt{(2m_{\text{Qd}}^{*}/\hbar^{2}){\left(R\right)}^{2}E}$. This equation can be solved using a Newton-Raphson algorithm that gives us a discrete energy levels *ϵ*_*i*_′. Since in the Schrödinger equation the zero energy origin is located at the bottom of the well, we need to shift the energy *ϵ*_*i*_′ in order to have a common Fermi level. We obtain

(10) $$\begin{array}{c} \rho {\left(E\right)}_{i}^{\text{CB}}=\overset{n}{\underset{i}{\sum }}\delta \left(E-E_{\text{displ}}-\epsilon_{i}^{\prime }\right), \end{array}$$

where *n* is the number of bounding states in the *i*th Qd. The value of *E*_displ_ is half the size of the bulk material gap where we assume the Fermi level is placed. Now, we define *ϵ*_*i*_ = *ϵ*_*i*_′ + *E*_displ_. Similar treatment is done for the hole binding states of the Qd.

Up to now we treated each Qd as an independent part of the system, but the Qds are coupled between them. This effect is introduced assuming a broadening of the discrete energy levels of the Qds. The standard way to introduce the broadening of the energy levels as a consequence of contacts is to assign a Lorentzian shape to each discrete energy level [42]:

(11) $$\begin{array}{c} \delta \left(E-\epsilon \right)⟶\frac{\gamma /2\pi }{{\left(E-\epsilon \right)}^{2}+{\left(\gamma /2\right)}^{2}}, \end{array}$$

where *γ* is the broadening of the level and it is related with the tunnel probabilities. Therefore, the total DOS for each Qd is the total sum of the energy levels taking into account the electron and hole binding states:

(12) $$\begin{array}{c} \rho_{i}=\overset{n}{\underset{i}{\sum }}\frac{\gamma /2\pi }{{\left(E-\epsilon_{i}\right)}^{2}+{\left(\gamma /2\right)}^{2}}. \end{array}$$

We have used a simplified model in order to describe the DOS structure of the Qds but the proposed approach allows to use more complicated DOS obtained using* ab initio* models. Therefore, this model is suitable to study the transport properties for several Qds materials and taking advantage of the atomistic theories as we have demonstrated in [43].

### 2.3. Potential Profile

Up to now we have only computed the nonequilibrium distribution function of electrons inside each Qd. Therefore, the Qds can be charged (or loose their charge) and these charge variations will affect the local potential of each Qd (*V*_*i*_). As we can see in Figure 1(c) each junction is modeled as a current tunnel junction in parallel with a capacity. These capacities represent the electrostatic influence between the different parts of the system. Therefore, each Qd has a local potential due to the applied bias voltage. Since each Qd can be charged, we need to solve the Poisson equation:

(13) $$\begin{array}{c} \vec{\nabla }\cdot \left(\varepsilon_{r}\vec{\nabla }V_{i}\right)=-\frac{q\Delta N_{i}}{\Omega }, \end{array}$$

where *ε*_*r*_ is the relative permittivity of the dielectric media and *Ω* is the Qd volume. Δ*N*_*i*_ is the change in the number of electrons, calculated with respect to the number of electrons *N*_0_ initially in the *i*th Qd. The potential energy of each Qd is *U*_*i*_ = −*qV*_*i*_. The inclusion of the charge term takes into account the carrier interaction inside each Qd. The result of this approach corresponds to the Hartree-term, that is, first approximation of the carrier-carrier interaction using a mean-field level treatment [44].

The general solution of the potential energy of the *i*th Qd involves the different capacitive coupling between it and its surrounding and its charge increasing [45] and it can be written as

(14) $$\begin{array}{c} U_{i}=\underset{j\neq i}{\sum }\frac{C_{ij}}{C_{\text{tot},i}}\left(-qV_{j}\right)+\frac{q^{2}}{C_{\text{tot},i}}\Delta N_{i}, \end{array}$$

where the subscript *j* runs over all the components of the system, *C*_*ij*_ is the capacitive coupling between the different components, and *C*_tot,*i*_ = ∑_*j*,*j*≠*i*_ *C*_*ij*_ is the total capacitive coupling of *i*th Qd. The charge energy constant *U*_0*i*_ = *q* ^2^/*C*_tot,*i*_ is the potential increase as a consequence of the injection of one electron into the Qd. Equation (14) is a set of equations (one equation per dot) and the first term of (14), the Laplace term *U*_*i*_^*L*^, can be written in a matrix form as

(15) U1L⋮UNL=1Ctot,1000⋮00⋯1Ctot,N×C1Lead⋮CNLead−qVds0C1,2⋯C1,NC2,10⋯C2,N⋮⋮⋮⋮CN,1CN,2⋯0hhh−0C1,2⋯C1,NC2,10⋯C2,N⋮⋮⋮⋮CN,1CN,2⋯0qV1⋮qVN.

The first term of the previous equation is the electrostatic influence of the lead in which the bias voltage (*V*_*ds*_) is applied; meanwhile, the second term is the electrostatic coupling with the neighbor Qd. The neighbors capacitive matrix is defined as *N* × *N* symmetric matrix with zero in the diagonal terms. Both terms are multiplied by the inverse of the total Qd capacity.

The effects of the local potential on each Qd should be computed in the Qd DOS *ρ*_*i*_(*E*) → *ρ*_*i*_(*E* − *U*_*i*_) shifting the position of the energy levels. This fact modifies the Qd charge and the currents. In (14) we observe that the local potential depends on the increasing charge density but at the same time the charge depends on the DOS which is modified by the local potential. These considerations impose a self-consistent solution of (5) and (14). The self-consitent solution of the system can be summarized as follows.

1. For a given external bias voltage, (6) are solved and the nonequilibrium distribution functions (*n*_*i*_) of each Qd are obtained. The Qds charge are evaluated as *N*_*i*_ = ∫*ρ*_*i*_(*E*)*n*_*i*_(*E*)*dE*.
2. The local potential in each Qd is obtained using (14).
3. The DOS of the Qds is shifted according to their respective local potential *ρ*_*i*_(*E*) → *ρ*_*i*_(*E* − *U*_*i*_). The transmission coefficients also change.
4. Repeat until the potential of the Qds converge.

Before we present in detail the code implementation, the capacitive couplings among the different parts are described.

#### 2.3.1. Capacitive Elements

A realistic modelization of the capacitive coupling between the different parts of the systems [46] is needed, since the electron needs available states in the Qds in order to have transport and the DOS of each Qd depends on the local potential. Therefore, the position of the energy levels with the applied bias voltage plays an important role in the determination of the *I*-*V* curve.

We use the analytical relationship for a sphere to conduct plane capacitance to model the capacitance between the leads and the Qd, which is

(16) $$\begin{array}{c} C_{i}^{\text{Lead}}=4\pi \varepsilon_{r}\sqrt{r^{2}-R^{2}}\overset{\infty }{\underset{n=1}{\sum }}\frac{1}{\sinh\left(n\;\text{arccosh}\left(r/R\right)\right)}, \end{array}$$

where *ε*_*r*_ is the permittivity of the dielectric media, *R* is the Qd radius, and *r* is the distance between the plane and the center of the Qd.

**Figure 3:**
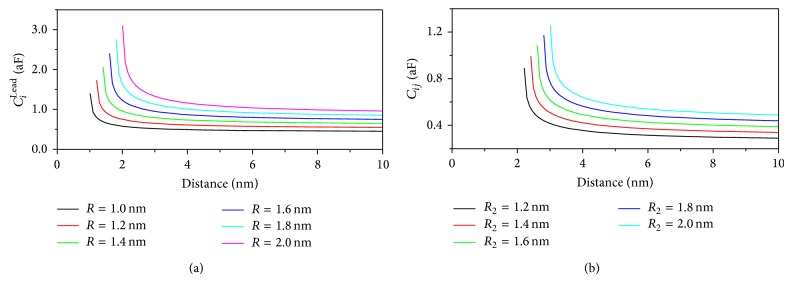
(a) Electrode-Qd capacity (*C*_*i*_^Lead^) for different Qds radii as a function of the distance. (b) Qd-Qd capacity (*C*_*ij*_) for different *R*_2_ radii; the radius of one Qd is hold at *R*_1_ = 1 nm. In both plots we use *ε*_*r*_ = 3.9*ε*_0_, where *ε*_0_ is the vacuum permittivity.

For the case of interdot capacitances (*C*_*i*,*j*_) there is no analytical expression for the capacitance that takes into account Qds of different radii. We use the numerical method of image charges to calculate interdot capacitance between Qds of different sizes. In Figure 3 we show the two capacitive terms as a function of the distance and for different Qd radii.

#### 2.3.2. Code Implementation

**Figure 4:**
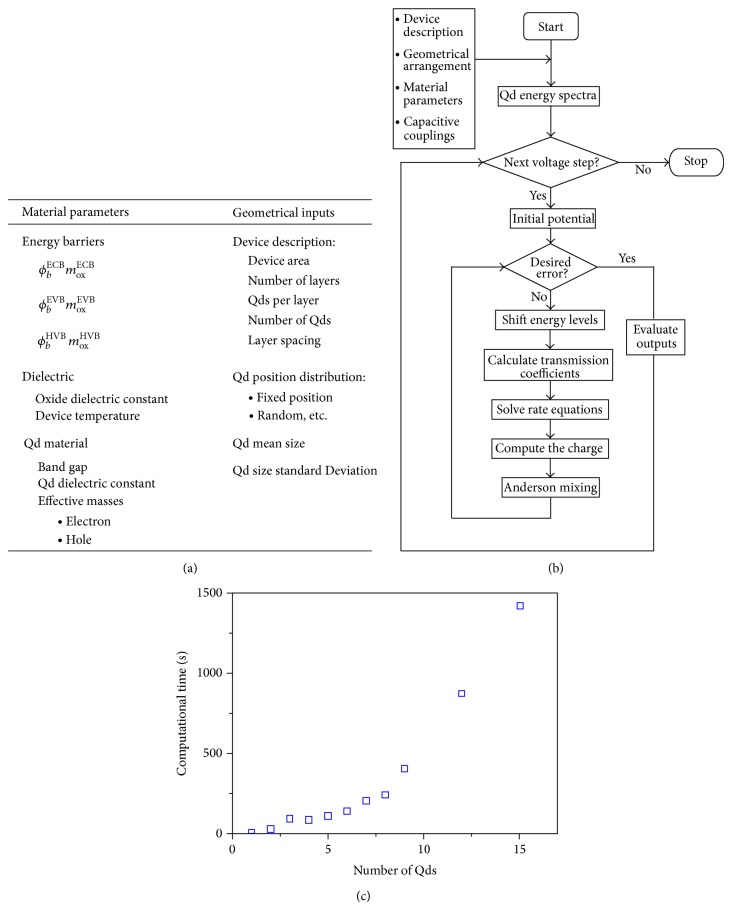
(a) List of the input parameters that describe the device. (b) Scheme flowchart of the self-consistent transport methodology. (c) Computational time versus number of simulated Qds. The time is referred for a single voltage point.

Since the methodology was presented and realistic parameters have been used, putting all together, we can describe the ballistic transport through an array of Qds embedded in an insulator matrix. We have to note that the inputs for the transport code are only material parameters and the geometry of the device. A summary of the different inputs is shown in Figure 4(a).

Now, we are going to describe briefly the code implementation and some computational strategies that we have used in order to create the computational tool. The code is divided in 3 main parts.

1. *Input parameters:* define the geometrical and the material parameters that form the device. The number of Qds is also defined. Calculate all the voltage independent parameters.
2. *SCF process:* start the voltage loop. For each voltage point, the SCF process is repeated until it converges to the desired error.
3. *Output:* calculate the output values.

The scheme flowchart of the code is presented in Figure 4(b). The first part of the code generates the system, the Qd arrangement, and takes into account the material parameters needed to describe the Qds and the insulator matrix. Moreover, the capacitive couplings, the binding electron/hole energy levels, and the tunneling distances are also calculated.

For each external bias voltage point, the self-consistent methodology is used to obtain a simultaneous convergence of the local potential and the charge in each Qd. In order to accelerate the convergence of this set of equations, we have implemented Anderson's method [47, 48]. This procedure consists of the minimization of the “distance” between the input (*U*_in_) and the output (*U*_out_) potentials. The distance is defined as

(17) $$\begin{array}{c} D\left[U_{\text{out}},U_{\text{in}}\right]={\left(\langle U_{\text{out}}-U_{\text{in}}\;|\;U_{\text{out}}-U_{\text{in}}\rangle \right)}^{1/2}={\langle F\;|\;F\rangle }^{1/2}, \end{array}$$

where 〈*U*_out_ − *U*_in_| is a vector of the differences for each Qd. Two “average” mixed potentials are defined for each iteration step as

(18) $$\begin{array}{c} |{\overset{-}{U}}_{\text{in}\left(\text{out}\right)}\rangle =\left(1-\beta \right)|U_{\text{in}\left(\text{out}\right)}^{\left(m\right)}\rangle +\beta |U_{\text{in}(\text{out})}^{\left(m-1\right)}\rangle , \end{array}$$

where *m* is the current iteration. The aim is to obtain the “best” *β* value for the current iteration which minimize the distance between these two average quantities. It reads as

(19) $$\begin{array}{c} \beta =\frac{\langle F^{\left(m\right)}\;|\;F^{\left(m\right)}-F^{\left(m-1\right)}\rangle }{D^{2}\left[F^{m},F^{m}\right]}. \end{array}$$

Finally, to obtain the new guess for the next iteration, we simply mix the average ${\overset{-}{U}}_{\text{in}}$ and ${\overset{-}{U}}_{\text{out}}$ potentials:

(20) $$\begin{array}{c} |U^{\left(m+1\right)}\rangle =\left(1-\alpha \right)|{\overset{-}{U}}_{\text{out}}^{m}\rangle +\alpha |{\overset{-}{U}}_{\text{in}}^{m}\rangle , \end{array}$$

where *α* is chosen empirically. This scheme is implemented in the code as follows.

1. Initialize the variables with the last value of the previous voltage point. For the first voltage point the variables start with zero.
2. Calculate the solution of the Poisson equation *U*_out_ for given *U*_in_.
3. Calculate the value of *β*.
4. Calculate the average mixing for the input ${\overset{-}{U}}_{\text{in}}$.
5. Calculate the average value for the output ${\overset{-}{U}}_{\text{out}}$.
6. Do the simple mixing between the input and output potentials.
7. Save values for the next iteration.
8. Repeat steps (2)–(7) until the desired convergence is achieved.

Once the convergence has been achieved, the outputs can been obtained for this voltage step. In a final staged part, the outputs (Qd occupancy versus voltage, current versus voltage, and local potential versus voltage) are calculated for each voltage point and saved in a matrix structure. The process repeats until all the bias voltage steps have been done using the previous potential results as the initial guess for the next bias voltage iteration. In Figure 4(c) we show the computational time needed to obtain results for one voltage point as a function of the number of Qds. The computational time grows with the number of Qds but it is still reasonable and allows simulating large Qd arrays.

The methodology has been explained in depth to enable the interested reader to create his/her own code and reproduce the following results. However, implementation of the code for specific devices is available (The developed code is available under agreement in contact with the author sillera@el.ub.edu).

## 3. Application and Discussion

The above-described self-consistent transport simulator has been applied to study the ballistic electronic transport in different generic devices based on silicon Qds embedded in SiO_2_ matrix (Si/SiO_2_ Qds). The parameters used to describe the materials are listed in Table 1. The *m*_ECB_^*^, *m*_EVB_^*^, and *m*_HVB_^*^ are the oxide effective masses for the different tunneling processes; the values are extracted from Lee and Hu [39]. *m*_Qd,CB_^*^ and *m*_Qd,VB_^*^ are the electron and hole Si bulk effective masses used to obtain the binding states in the Qd. We consider that the equilibrium Fermi level is in the middle of Si *E*_gap⁡_; confinement potentials of the Qd are *ϕ*_1,ECB_ for electrons and *ϕ*_1,HVB_ for holes, respectively. Finally, *ε*_*r*_ is the relative permittivity of the SiO_2_ matrix.

The electron transport takes place from the left electrode to the right electrode through the Qds. From a physical point of view, the most general transport condition is that the energy levels of the Qds (*ϵ*_*i*_) must lie between the electrochemical potentials of the leads (*μ*_*L*_ − *μ*_*R*_ = −*qV*_*ds*_); this condition can be summarized as *μ*_*L*_ ≥ *ϵ*_*i*_ ≥ *μ*_*R*_. The type of transport will depend on the nature of these energy levels and electron or hole binding energy levels. Moreover, in order to have transport between the Qds, overlapping of the Qd DOS is necessary. Free states in the arriving Qd are also needed. These two conditions are directly related to the expression of the tunneling current described by (4). The transmission coefficients are also strongly dependent on the tunneling distance; therefore, some processes are more favored than others. Thus, the electronic transport plays with the transmission probabilities between the different processes and the available states.

The total net current will be the sum of the partial tunnel currents among the Qds and the right lead (electron and hole terms). This net current is going to be dependent on the position of the Qds, the tunneling distances, and the alignment of the energy levels (the local potential and the DOS of each Qd).

### 3.1. Single Si/SiO_2_ Qd

Before we simulate a complete device based on large arrays of Qds, we are going to describe a single Si/SiO_2_ Qd in different configurations. From this small system, we will show the basis of the ballistic transport and the effects of the geometrical arrangement of the Qds in the final electrical response.

**Figure 5:**
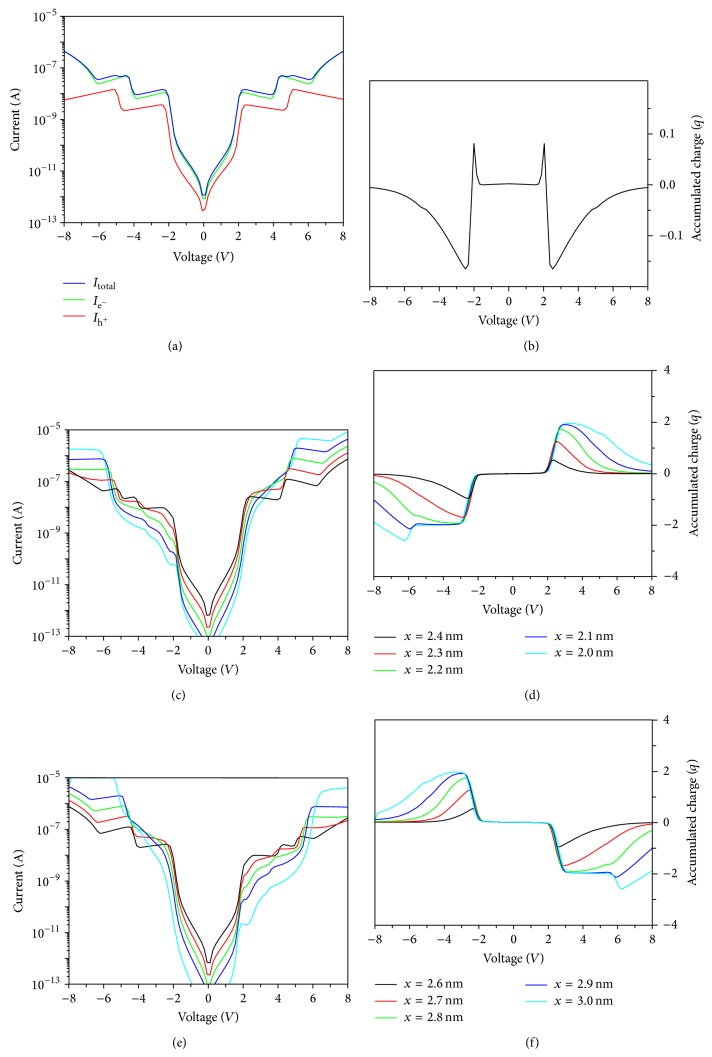
A single Si/SiO_2_ Qd of *R* = 1.5 nm placed in different positions between the two leads. *x* is the distance from the left lead to the center of the Qd. The separation among the leads is 5 nm. ((a)-(b)) *I*(*V*) curve and accumulated charge for a centered Qd. The hole and electron currents are also shown. ((c)–(e)) *I*(*V*) curves for different Qd positions and ((e)-(f)) accumulated charge in the same cases.

In Figure 5, we present the obtained *I*(*V*) curves and the accumulated charge for a system composed of two leads separated by 5 nm and a single Si/SiO_2_ Qd connected to them placed at different positions. The position of the Qd, *x*, is measured from the left lead. An external bias voltage is applied to the right lead whereas the left one is kept as a reference; this means *μ*_*L*_ = 0 and *μ*_*R*_ = −*qV*. Concerning the accumulate charge, it reflects the variation of the number of electrons with respect to the initial number. Therefore, if the charge is negative it implies that the system looses charge (hole accumulation). However, if the charge is positive this implies that the system increases its charge (electron accumulation).

Figure 5(a) shows the total *I*(*V*) curve and the hole and electron currents for a Qd connected symmetrically to both leads, which is *x* = 2.5 nm. Concerning the partial currents (electron and hole contributions), the electron current is the dominating term since the electron barrier (3.1 eV) is smaller than the hole barrier (4.5 eV). Besides, the opening of the discrete electron/hole conductive channels is clearly visible in the current steps at different voltages due to the position of the discrete electron/hole energy levels. Since the system is symmetrically coupled to the leads, the total current is symmetric in both polarization directions.

Regarding the accumulated charge, in Figure 5(b), the Qd remains practically uncharged. The obtained trend reflects the position of the electron and hole energy states respect to the equilibrium Fermi level and the effect of the self-charge. An electron state is the first energy level that starts to be conductive but a hole conductive channel is opened immediately being the Qd accumulated charge the difference between the electron and hole fluxes.

The *I*(*V*) curves and the accumulated charge are shown in Figures 5(c)-5(d) and 5(e)-5(f) for the nearest case to the left lead (*x* < 2.5) and for the right lead (*x* > 2.5), respectively. As can be seen, the symmetry in the total *I*(*V*) curve has been broken due to the different capacitive coupling among the leads [49]. Moreover, the Qd gains/loses net charge. Besides, we must note that the obtained trends for the *x* < 2.5 cases are complementary to the *x* > 2.5 cases.

When an external polarization is applied, different incoming/outgoing fluxes are created and the occupation of the states differs from the equilibrium case. Besides, the transport takes place on the energy region between *μ*_*L*_ and *μ*_*R*_. Thus, only the energy states placed in this energy region can gain or lose charge. From the rate equation model, the Qd nonequilibrium distribution function in the steady state can be viewed as a balance between the two leads that strongly depends on the transmission coefficients and the occupation of the leads. Since the leads occupation is well described by the electrochemical potentials *μ*_*L*/*R*_, the Qd energy level occupation is dominated by the highest transmission coefficient. Therefore, the lead connected to the Qd with the highest transmission coefficient dominates the Qd occupation. Since the transmission coefficients are strongly dependent on the tunneling distance, for lower voltages, the closer lead will dominate the final response. However, for larger voltages, the band bending of the wider oxide increases giving an smaller effective tunneling distance and the Qd recovers its initial charge.

### 3.2. Electron Transport through Si/SiO_2_ Qds Arrays

In order to deal with devices based on Qd arrays, we are going to study the electron transport in a multilayer structure. This part is going to be one of the most important building blocks of the devices and, therefore, it is important to have a good characterization of it. From the experimental point of view, the superlaticce approach (SL) [50–52] allows creating these kind of structures: layers of Qds separated by insulator layers and size-controlled Qds.

**Figure 6:**
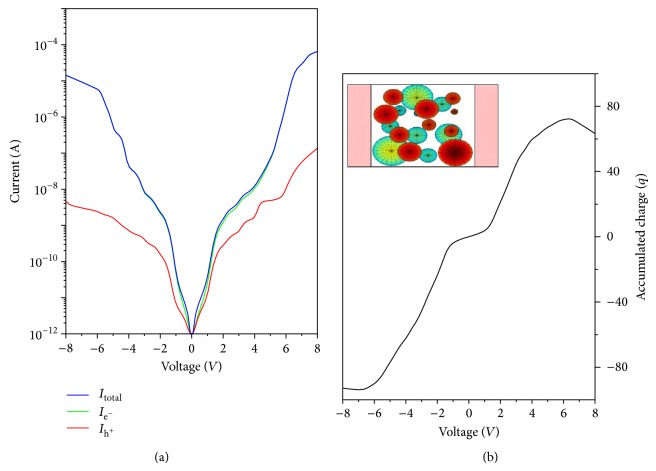
(a) Obtained *I*(*V*) curves (electron/hole and total currents) for two layers of 10 Si/SiO_2_ Qds. The system arrangement is described in the text. The Qds radius distribution is also shown in the inset. (b) Total accumulated charge (∑_*i*_Δ*N*_*i*_ where *i* = 1 ⋯ 20) of the structure as a function of the external bias voltage. In the inset, a top view of the system is presented.

Here, we present the *I*(*V*) and the total accumulated charge for a multilayer structure. The structure is formed by 2 Si Qds layers. The leads are placed perpendicular to them; therefore, the electronic transport takes place laterally. Both layers are spaced by 5 nm and the layer size is 20 nm length and 20 nm width. We simulate 10 randomly distributed Qds per layer and for the Qds radii we use a normal distribution with 1.75 nm mean value and 0.3 nm standard deviation. A top view scheme of the system is shown in the inset of Figure 6(b).

In Figure 6(a), the total *I*(*V*) curve and the electron and hole currents terms are shown. From the *I*(*V*) curves, two different regimes are obtained: for low and medium voltage ranges, the current reflects the discrete nature of the Qds energy spectra. However, for the largest voltages, the continuous part of the Qd DOS begins to be conductive and the current increases in a continuous form loosing its step-like shape. Concerning the electron/hole current components, the electron term dominates since its potential barrier is lower than the hole one. Moreover, the stepping behavior is still present in the current reflecting the aperture of the different conductive channels. In this case, the transport involves several tunneling processes between the Qds. Therefore, the energy level alignment is necessary. However, the negative differential resistance (NDR) is not clearly visible since there are many electron/hole pathways and the sum of the different conduction channels masks this effect [22].

Regarding the accumulated charge plotted in Figure 6(b), it is the total sum of the accumulated charge in each Qd (∑_*i*_Δ*N*_*i*_ where *i* = 1 ⋯ 20). The geometrical disposition of the Qds strongly affects the final response of the system as we have shown in the previous section. For this system, since there are many electronic conduction channels and a complex capacitive interaction between the Qds, it is hard to explain clearly the accumulated charge trend. Reflecting that, the geometrical disposition of the Qds influences directly the final response of the system.

## 4. Conclusions

The high efficiency concepts of the next generation of Qds based devices pose new requirements on models for the theoretical description of their transport properties. An intuitive theoretical framework suitable for this purpose is available in the noncoherent rate equations. This approach provides a simple and transparent method to describe the electron transport. Using the Transfer Hamiltonian approach to describe the tunneling current terms in ballistic regime, the rate equations can be used in order to obtain the nonequilibrium distribution functions in each Qd. The effect of self-charge has been taken into account solving the Poisson equation with the appropriate boundary conditions for each Qd that involve the capacity coupling between the different parts of the system and the accumulated charge in each Qd. As expected, the calculation of the local potential inside each Qd is one of the most critical points, since the *I*-*V* curves depend on the position of the energy level. Due to the simplicity of the model, this can be easily extended to analyze arbitrary large arrays of Qds of interest in technological applications creating a powerful and useful tool that enables to design new concept devices based on Qd properties.

In order to simulate devices as realistic as possible, suitable expressions for the transmission coefficients, the energy level positions and the capacitive coupling have been used. These parameters can be described using basic material properties and geometrical representations of the system. Moreover, the hole currents have been taken into account, obtaining a complete description of the electron transport in the structure.

Finally, the proposed formalism has been used to describe, using realistic material parameters, the electrical response of a single Si/SiO_2_ Qd. Moreover, a bilayer structure has also been simulated. This structure composes the basic building block for future devices based on Qds and demonstrates the practicability of the here presented approach.

## Figures and Tables

**Table 1:** Parameters used in the simulation in order to describe Si Qds embedded in SiO_2_ insulator matrix. *m*_0_ and ε_0_ are the free electron mass and the vacuum permittivity, respectively.

*m*_ECB_^*^ (*m*_0_)	0.40 [23, 24]	ϕ_1,ECB_ (eV)	3.1 [25, 26]
*m*_EVB_^*^ (*m*_0_)	0.30 [23]	ϕ_1,HVB_ (eV)	−4.5 [25, 26]
*m*_HVB_^*^ (*m*_0_)	0.32 [23]	*E*_gap⁡_ (eV)	1.12 [25, 26]
*m*_Qd,CB_^*^ (*m*_0_)	0.33 [27]	ε_*r*__SiO_2__ (ε_0_)	3.9 [26, 28]
*m*_Qd,VB_^*^ (*m*_0_)	0.28 [27]	ε_*r*__Si_ (ε_0_)	11.7 [26, 28]
